# Assessment of glucose-6-phosphate dehydrogenase activity using CareStart G6PD rapid diagnostic test and associated genetic variants in *Plasmodium vivax* malaria endemic setting in Mauritania

**DOI:** 10.1371/journal.pone.0220977

**Published:** 2019-09-16

**Authors:** Oum kelthoum Mamadou Djigo, Mohamed Abdallahi Bollahi, Moina Hasni Ebou, Mohamed Salem Ould Ahmedou Salem, Rachida Tahar, Hervé Bogreau, Leonardo Basco, Ali Ould Mohamed Salem Boukhary

**Affiliations:** 1 Unité de recherche Génomes et Milieux, Faculté des Sciences et Techniques, Université de Nouakchott Al-Aasriya, Nouveau Campus Universitaire, Nouakchott, Mauritania; 2 Centre National de Transfusion Sanguine, Ministère de la Santé, Nouakchott, Mauritania; 3 UMR 216 MERIT, IRD, Faculté de Pharmacie, Univ. Paris Descartes, Paris, France; 4 Unité de Parasitologie et d’Entomologie, Institut de Recherche Biomédicale des Armées, IHU-Méditerranée Infection, Marseille, France; 5 Aix Marseille Univ, IRD, AP-HM, SSA, VITROME, Marseille, France; 6 IHU-Méditerranée Infection, Marseille, France; 7 Centre National de Référence du Paludisme, Institut Hospitalo-Universitaire (IHU) Méditerranée Infection, Marseille, France; Universidade Nova de Lisboa Instituto de Higiene e Medicina Tropical, PORTUGAL

## Abstract

**Background:**

Primaquine is recommended by the World Health Organization (WHO) for radical treatment of *Plasmodium vivax* malaria. This drug is known to provoke acute hemolytic anemia in individuals with glucose-6-phosphate dehydrogenase (G6PD) deficiency. Due to lack of data on G6PD deficiency, the use of primaquine has been limited in Africa. In the present study, G6PD deficiency was investigated in blood donors of various ethnic groups living in Nouakchott, a *P*. *vivax* endemic area in Mauritania.

**Methodology/Principal findings:**

Venous blood samples from 443 healthy blood donors recruited at the National Transfusion Center in Nouakchott were screened for G6PD activity using the CareStart G6PD deficiency rapid diagnostic test. G6PD allelic variants were investigated using DiaPlexC G6PD genotyping kit that detects African (A^-^) and Mediterranean (B^-^) variants. Overall, 50 of 443 (11.3%) individuals (49 [11.8%] men and 1 [3.7%] woman) were phenotypically deficient. Amongst men, Black Africans had the highest prevalence of G6PD deficiency (15 of 100 [15%]) and White Moors the lowest (10 of 168, [5.9%]). The most commonly observed G6PD allelic variants among 44 tested G6PD-deficient men were the African variant A^-^ (202A/376G) in 14 (31.8%), the Mediterranean variant B^-^ (563T) in 13 (29.5%), and the Betica-Selma A^-^ (376G/968C) allelic variant in 6 (13.6%). The Santamaria A^-^ variant (376G/542T) and A variant (376G) were observed in only one and two individuals, respectively. None of the expected variants was observed in 8 (18.2%) of the tested phenotypically G6PD-deficient men.

**Conclusion:**

This is the first published data on G6PD deficiency in Mauritanians. The prevalence of phenotypic G6PD deficiency was relatively high (11.3%). It was mostly associated with either African or Mediterranean variants, in agreement with diverse Arab and Black African origins of the Mauritanian population.

## Introduction

Malaria remains a major public health burden in Mauritania with two-thirds of the population at risk. In the northern Saharan zone of the country, including Nouakchott, the capital city, malaria is predominantly caused by *Plasmodium vivax* [1, 2], whereas in the southern Sahelian zone, almost all malaria cases are due to *Plasmodium falciparum* [3, 4].

*Plasmodium vivax* infection is characterized by the occurrence of relapses several weeks or even months after the primary infection due to the persistent quiescent liver forms (hypnozoites). Radical cure of *P*. *vivax* malaria requires the elimination of both the blood stage parasites and hypnozoites. Primaquine, an 8-aminoquinoline, has long been the only available and effective drug capable of killing hypnozoites and, therefore, of preventing relapses [5]. Tafenoquine (Krintafel), another 8-aminoquinoline with a hypnozoicidal activity, has recently been approved by the United States Food and Drug Administration [6]. Despite their effectiveness in eliminating hypnozoites, 8-aminoquinolines may cause dose-dependent oxidant hemolysis in glucose-6-phosphate dehydrogenase (G6PD) deficient individuals [7, 8].

G6PD deficiency is the most common genetic enzyme disorder in humans with 400 million affected persons worldwide. Currently, 217 mutations on the *g6pd* gene have been described and genetically characterized [9, 10]. In sub-Saharan Africa, the most frequent G6PD variants are the wild-type G6PD B, the non-deficient variant G6PD A, and the deficient variant G6PD A^-^ [11]. The variant G6PD A results from the point mutation A376G whereas the deficient variant G6PD A^-^ is characterized by the combination of A376G mutation and one of the following mutations: G202A, A542T, G680T, or T968C. In North Africa, *g6pd* gene with a point mutation (C563T), known as the “Mediterranean variant (B^-^),” also occurs [12]. The G6PD A^-^ variant is often associated with mild to moderate hemolytic anemia, as compared to other G6PD variants, although fatal reactions to primaquine have been recorded in a limited number of patients [13]. The Mediterranean variant often results in a more clinically severe hemolytic reaction following primaquine therapy and predisposes individuals to favism [14]. Because of its X-linked mode of inheritance, G6PD deficiency affects men more frequently than women.

Current G6PD deficiency diagnostic methods include the fluorescent spot test [15], spectrophotometric assay, also called enzyme activity assay [16], and cytochemical assay [17]. Although these techniques are reliable to identify G6PD deficiency, they are time consuming and require sophisticated laboratory equipment and skilled personnel to perform the tests, making them difficult to use in low-resource settings [18]. Recently, several qualitative rapid diagnostic tests for screening this enzymatic disorder have been developed [19]. These provide the results rapidly (within 10–15 min), work in a wide range of ambient temperature, use small amounts of fresh whole capillary blood, and are not costly, compared to conventional diagnostic tests performed in well-equipped laboratories [19, 20, 21].

Since 2014, primaquine is recommended by the Mauritanian Ministry of Health for the radical treatment of *P*. *vivax* malaria in patients with normal G6PD activity [22]. Before primaquine can be administered to a patient for radical treatment of *P*. *vivax* malaria, screening for G6PD activity is required because primaquine is contraindicated in patients with G6PD deficiency. However, in almost all health facilities in the country, the G6PD status of *P*. *vivax*-infected malaria patients is not or cannot be determined at present, leading to non-use of primaquine. Moreover, there is no recent database on G6PD status in Mauritania. Therefore, the objective of this study was to assess the prevalence of different variants of G6PD deficiency in Mauritanian blood donors of different ethnic origins in Nouakchott, where *P*. *vivax* is endemic. The obtained data will be useful for decision making on the possible need for routine G6PD deficiency testing as part of *P*. *vivax* malaria control and elimination in the country.

## Methods

### Study site

**Fig 1:**
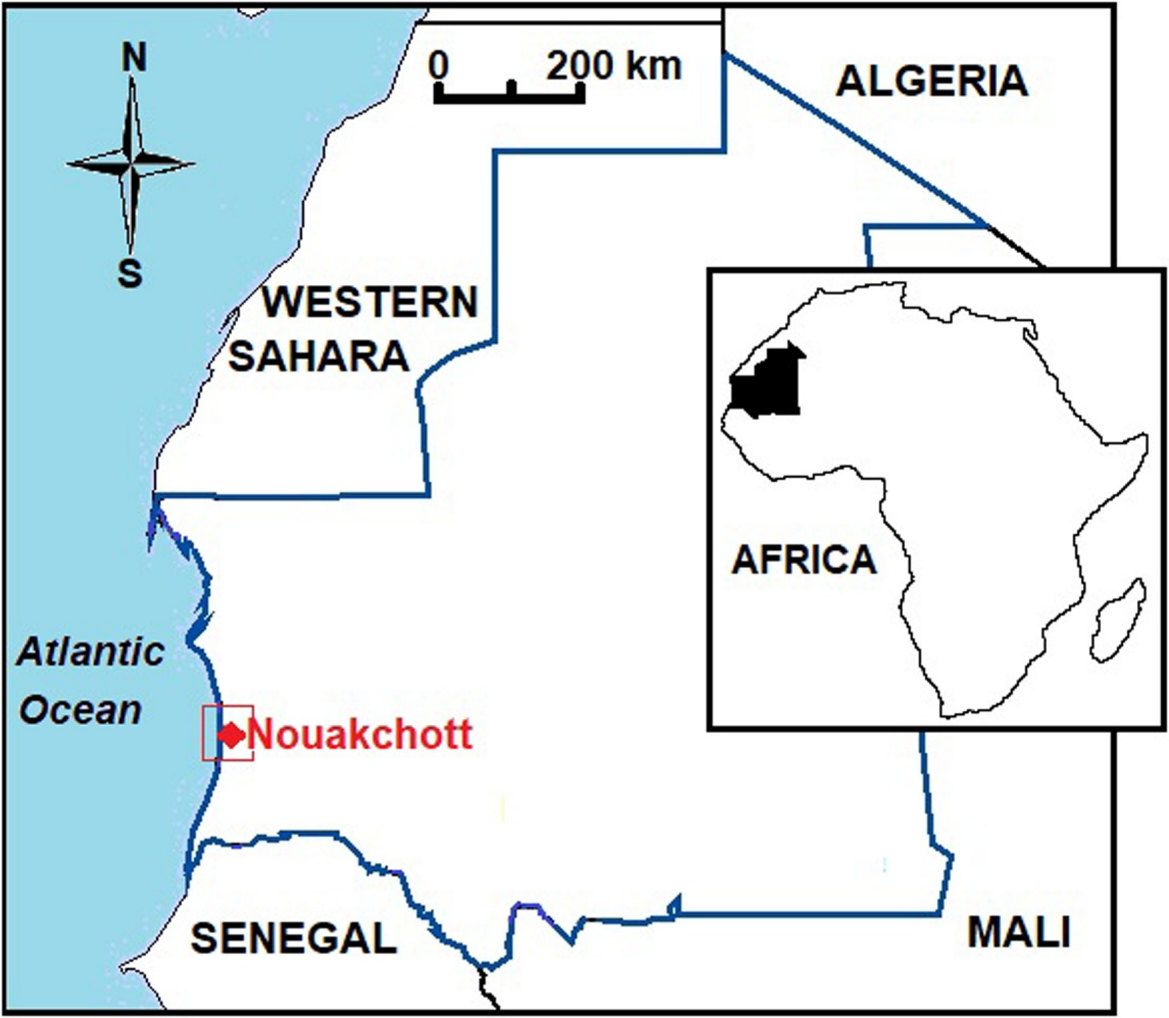
Study site for the investigation of G6PD deficiency in Mauritania (red box). Inset map shows location of Mauritania in Africa. This map was created using Microsoft Paint application (Redmond, WA).

The study was conducted in 2016 at the National Blood Transfusion Center (CNTS) in Nouakchott (Fig 1). The Nouakchott that contributes to a quarter of the total population (1,043,177) of Mauritania reflects the diverse ethnicity of the country [23]. The two major ethno-linguistic groups are the Moors (consisting of white and black Moors) and black Africans (including Pular, Soninke, and Wolof). White and black Moors share the same dialect called “*Hassaniya*” derived from a Berber-influenced Arabic language, while each of Black African ethnic groups has its own language and cultural specificities [24]. Inter-ethnic marriages are rare in Mauritania.

### Blood collection and detection of G6PD deficiency

Between May and December 2016, whole blood was collected in a 5-ml ethylenediaminetetraacetic acid (EDTA)-coated blood collection tube from 454 healthy blood donors and was immediately used for G6PD deficiency screening using CareStart rapid diagnostic test (AccessBio, Somerset, NJ, USA) according to the manufacturer’s instructions. About 200 μl of the remaining blood were spotted on Whatman 3MM filter paper for storage and DNA extraction.

### DNA extraction and detection of G6PD allelic variants

DNA was extracted from samples with G6PD deficiency using Quick-DNA Miniprep Plus kit (Zymo Research Corporation, Irvine, CA, USA) according to the manufacturer’s instructions. G6PD variants were characterized using DiaPlexC G6PD genotyping kit (SolGent Co., Daejeon, Republic of Korea). The kit provides a simple and rapid alternative to PCR and enzyme digestion-based assays and enables to detect six different *g6pd* point mutations by a one-step multi-allelic specific PCR [10, 25]: G202A (Val-to-Met substitution in exon 4), A376G (Asn-to-Asp substitution in exon 5), A542T (Asp-to-Val substitution in exon 6), C563T (Ser-to-Phe substitution in exon 6), G680T (Arg-to-Leu substitution in exon 7), and T968C (Leu-to-Pro substitution in exon 9). These mutations are associated with three levels of deficiency according to the severity of clinical symptoms that may occur in the presence of stressors [10, 26]: class II (severe deficiency; Mediterranean B^-^, C563T single mutation; Santamaria, A376G + A542T double mutations), class III (mild to moderate deficiency; A^-(202)^, G202A + A376G double mutations; A^-(680)^, A376G + G680T double mutations; A^-(968)^ Betica-Selma, A376G + T968C double mutations), and class III-IV (mild deficiency; type A, A376G single mutation).

Two microliters of total genomic DNA (25–50 ng) were added to 23 μl of master mixture containing pre-mix (12.5 μl), G6PD primer mixture (2 μl; African type), and nuclease-free ultrapure water (8.5 μl), as recommended by the manufacturer. PCR was performed for 30 cycles at an annealing temperature of 60°C, as recommended by the manufacturer. PCR products were analyzed by electrophoresis on 3% agarose gel, stained with GelGreen Nucleic Acid Gel Stain (Biotium Inc., Fremont, CA, USA), and visualized under ultraviolet illumination. The following sizes of PCR products were obtained, depending on the presence of mutations: A376G, 103 bp; G202A, 157 bp; A542T, 241 bp; C563T, 220 bp; G680T, 388 bp; and T968C, 463 bp. The validity of each PCR amplification was confirmed by an internal control (947 bp). PCR was performed in a thermal cycler Eppendorf MasterCycler Personal (Eppendorf, Hamburg, Germany).

### Data analysis

Data were analyzed using MedCalc statistical software version 16.4.3 [27]. The chi-square test was used for comparison of proportions. The significance level was set at P < 0.05.

### Ethical approval

The study obtained ethical clearance from the ethical committee of the University of Nouakchott Al-Aasriya, and written informed consent was obtained from all participants.

## Results

### Characteristics of the study population

Overall, 454 unrelated individuals were enrolled during the study period. Data from 11 donors (2.4%) were excluded from analysis due to missing data (5 without ethnic origin, 2 without gender, 4 invalid G6PD screening tests). Data from the remaining 443 individuals were analyzed. Table 1 summarizes the characteristics of the study population. The median (range; interquartile range) age was 30 (18–61; 12) years. Individuals under 18 years old were not represented in our sample population as the lower age limit for blood donation in the country is 18 years. The proportion of male donors (416/443; 94%) was significantly higher (P < 0.0001) than that of females (27/443; 6%). All ethnic groups in Mauritania were represented in the study population (168 white Moors, 173 black Moors, 100 Black Africans, and 2 mixed ethnic origins). The proportion of white and black Moors in the study population did not differ significantly (P = 0.85).

**Table 1:** Demographic characteristics and G6PD-deficient phenotypes using Carestart rapid diagnostic test in healthy blood donors in Nouakchott, Mauritania.

Characteristics		N (%)
Total number of individuals		443
Gender		
	Male	416 (94)
	Female	27 (6)
Ethnic group		
	White Moors	168 (38)
	Black Moors	173 (39)
	Black Africans*	100 (22.5)
	Mixed	2 (0.5)
G6PD phenotype		
	Deficient	50 (11.3)
	Normal	393 (88.7)
	Deficient, men	49 (11.8)
	Deficient, woman	1 (3.7)
G6PD deficiency and ethnic groups		
	White Moors	10 (5.9)
	Black Moors	25 (14.4)
	Black Africans**	15 (15)

*Pular, Soninke and Wolof;

**12 Pulars and 3 Soninkes

### Prevalence of G6PD deficiency

Among 443 screened study subjects, 50 (11.3%) were phenotypically deficient for G6PD using CareStart rapid diagnostic test (Table 1). G6PD deficiency was found, with no significant differences, in 49 of 416 (11.8%) males and 1 of 27 (3.7%) females (*χ*^2^ = 1.6; df = 1; P = 0.8). The prevalence of G6PD deficiency by ethnic groups indicated that Black Africans (15/100, 15.0%) and black Moors (25/173; 14.4%) had similar frequencies of G6PD deficiency (P = 0.89). Compared to these two ethnic groups, the prevalence of G6PD deficiency in white Moors was 5.9% (10/168; P = 0.009). Of 50 G6PD-deficient blood donors, 13 (26%) reported that they had a past history of malaria but were not able to remember the *Plasmodium* species and treatment they received.

### Identification of G6PD allelic variants

G6PD allelic variants were assessed in 45 (44 men and 1 woman) of 50 phenotypically G6PD-deficient individuals. PCR failed in 5 of 50 samples, probably due to poor storage conditions leading to DNA degradation. G6PD variant A (376G), characterized by little or no clinical significance, was observed in three individuals (two men and one woman) and was associated with a faint color change in CareStart rapid diagnostic test. Table 2 summarizes the results of molecular analysis in 44 phenotypically G6PD-deficient males. The most commonly observed G6PD allelic variant among G6PD deficient men was the moderate African variant A^-^ (202A/376G) in 14 of 44 (31.8%) individuals. Although the sample size was small, the African variant A^-^ was observed more frequently in Black Moors than the other ethnic groups (10 Black Moors vs. 3 Black Africans and 1 White Moor). By contrast, in this small sample of G6PD-deficient Mauritanians, the severe Mediterranean variant B^-^ (563T) was observed in 13 of 44 (29.5%) subjects and occurred more frequently in White Moors (8 White Moors vs. 3 Black Moor and 2 Black Africans). The moderate A^-^ (376G/968C) allelic variant (also known as Betica-Selma) was observed in 6 of 44 (13.6%) (5 Black Africans and 1 Black Moor) G6PD-deficient subjects. The severe Santamaria variant A^-^ (376G/542T) was observed in only one White Moor. None of the expected allelic variants detectable by DiaPlexC G6PD genotyping kit was observed in 8 (18.2%) of the tested phenotypically G6PD deficient men from two different ethnic groups (5 Black Moors and 3 Black Africans). The allelic variant G680T was not found in our study population.

**Table 2:** G6PD allelic variants among phenotypically deficient male blood donors by ethnic group in Nouakchott, Mauritania.

G6PD allelic variant*		Common name	Ethnic group			Total (%)
			White Moors	Black Moors	Black Africans**	
Single	376G	A	0	2	0	2 (4.5)
	563T	Mediterranean (B^-^)	8	3	2	13 (29.5)
Double	202A/376G	African (A^-^)	1	10	3	14 (31.8)
	376G/968C	Betica-Selma	0	1	5	6 (13.6)
	376G/542T	Santamaria	1	0	0	1 (2.3)
Uncharacterized***			0	5	3	8 (18.2)
Total			10	21	13	44 (100.0)

*PCR failed in samples from 5 phenotypically G6PD-deficient males, most probably due to DNA degradation;

** 11 Pular and 2 Soninke;

***None of the six tested allelic variants was detected in these individuals with phenotypic deficiency although the internal control was positive. The only woman with G6PD deficiency had G6PD A (376G) genotype. G680T was not found.

## Discussion

In countries where *P*. *vivax* is endemic, primaquine and more recently tafenoquine remain the only 8-aminoquinoline antimalarial drugs available for radical treatment. In Mauritania, several studies have confirmed that *P*. *vivax* is endemic in the northern Saharan zone, including Nouakchott [1–3]. White Moorish individuals are more susceptible to *P*. *vivax* infection than other ethnic groups, probably due to the high prevalence of positive Duffy blood group among them, even though *P*. *vivax* was also found in few Duffy-negative patients [1, 28, 29]. Primaquine was introduced in the national malaria treatment guideline in 2014 for the control and elimination of *P*. *vivax* malaria. At present, artemisinin-based combination therapy (ACT) is the first-line drug to treat *P*. *falciparum* and *P*. *vivax* malaria. Primaquine administration has not been implemented in the country owing to concerns about its potential toxicity, including hemolytic anemia, in G6PD-deficient patients and the absence of a prior routine G6PD deficiency testing for the safe use of primaquine at health facilities.

In the present study, G6PD deficiency was investigated in blood donors living in Nouakchott, an endemic area of *P*. *vivax* in Mauritania. Our results showed that the prevalence of phenotypic G6PD deficiency using CareStart rapid diagnostic test is 11.3%. The mean prevalence of G6PD deficiency among countries with ongoing malaria transmission is estimated to be 8% [9, 30]. The prevalence of G6PD deficiency reported in the present study is similar to those reported from other West African countries, such as Senegal (12%) [31], Sierra Leone (11.3%) [32] and Burkina Faso (9.5%) [33]. On the other hand, the prevalence of G6PD deficiency in Mauritania is higher than that reported in North African countries, such as Tunisia (4.4%), Algeria (5.4%), and Egypt (5.9%) [34, 35]. However, comparison of the frequency of G6PD deficiency (5.9%) in White Moors of Arab descent and that of the dominant Arab ethnic groups in Tunisia and Algeria reveals comparable results.

The study also revealed that the most common G6PD variants were G6PD A^-^ (African variant) and G6PD B^-^ (Mediterranean variant) found in 31.8% and 29.5% of G6PD-deficient blood donors, respectively. G6PD A^-^ and G6PD B^-^ are among the most common G6PD variants worldwide. They may be associated with different severity of clinical symptoms in the presence of antimalarial drugs such as primaquine, ranging from moderate (for G6PD A^-^) to severe (for G6PD B^-^) anemia. In sub-Saharan Africa, G6PD A^-^ (202A/376G) variant is the most common variant [14]. For instance, in Burkina Faso, A^-^ (202A/376G) was the only variant observed [33]. In Mali, A^-^ (202A/376G) was also observed but the differences in proportions between ethnic groups were not significant [36]. In some Arab African countries like Algeria and Tunisia, the frequency of G6PD A^-^ was as high as 46% [12, 35]. Inversely, in Senegal and The Gambia, A^-^ (376G/968C) was the most frequent allele associated with G6PD deficiency, with the prevalence of 10% and 7%, respectively [31, 37, 38]. The Mediterranean G6PD variant has not yet been described in sub-Saharan Africa, but it is the second most common variant in Algeria (23%) and Tunisia (33.1%) [12,35].

Eight of 44 phenotypically deficient male blood donors showed none of the six expected mutations. One possible explanation is that G6PD deficiency in these ethnically different individuals is associated with other variants not detected using DiaPlexC G6PD genotyping kit and that the observed phenotype could be associated with different G6PD genotypes. Only sequencing of *g6pd* gene in these individuals could provide further information. In Tunisia, no mutation was identified in 6 of 56 (10.7%) G6PD-deficient screened subjects using PCR-restriction fragment length polymorphism (RFLP) [39]. It is also possible that 8 G6PD-deficient subjects whose genotype was not characterized in our study could be false positives, but due to insufficient resources, we were not able to test the performance of CareStart rapid diagnostic test compared to the gold standard. Other studies have reported that diagnostic performance of CareStart rapid diagnostic test is high compared to other existing rapid diagnostic tests, and that this rapid diagnostic test is considered as a promising tool for point-of-care use in countries with poor resources [40–43]. In the previous studies, the sensitivity and specificity of CareStart rapid diagnostic test, compared to the reference quantitative spectrophotometric method, were 85–100% and 72–99%, respectively, at the threshold of 30% of normal G6PD activity [40–45]. Low sensitivity (62–68%) was reported in two studies, but the source of these discrepant results was due to the misinterpretation of faint color change which, if corrected, would have increased the sensitivity [46–48]. These data lend support to the view that this rapid diagnostic test may become an indispensable tool to screen *P*. *vivax*-infected patients before primaquine is administered.

Overall, the present study revealed heterogeneous prevalence of both African and Mediterranean variants of G6PD deficiency using phenotypic and molecular tests among study subjects belonging to different ethnic groups present in Mauritania. These findings were somewhat expected because Mauritania is located at the intersection between Maghreb and sub-Saharan African countries, and its ethnic composition is made up of Moors of Arab descent, in whom Mediterranean G6PD variant B^-^ (563T) predominates, and black Africans in whom G6PD deficiency is mostly due to G6PD A^-^ variant.

The main limitation of the present study was the inability to link the observed phenotypic and genotypic G6PD deficiency with enzymatic activity determined by a reference method in the study population. Moreover, despite the fact that 454 healthy individuals were recruited, the sample size of the present study does not have an adequate power to compare differences according to sex, ethnicity, and genotyping data. Nonetheless, to our knowledge, this is the first report on the prevalence of G6PD deficiency and its molecular variants in Mauritania. Further genotyping studies are needed to confirm the presence of additional variants that may be associated with G6PD deficiency and determine further the frequencies of mutations in G6PD in the general population as well as in different ethnic groups in relation to *P*. *vivax* infection. Accurate and up-to-date mapping of the prevalence of G6PD deficiency and its variants is required to design appropriate strategies of *P*. *vivax* malaria elimination that includes mass administration of primaquine.

## Conclusion

The observed prevalence of G6PD deficiency and molecular variants are in agreement with those observed in West and North African populations. The study highlights the need to implement adequate G6PD deficiency screening with a reliable rapid diagnostic method before prescribing primaquine for the radical treatment of *P*. *vivax*-infected patients in Mauritania to minimize the risk of clinically relevant and severe hemolytic anemia associated with different G6PD variants in the country. More studies on a larger population size, including a higher number of women, are being planned in areas where *P*. *vivax* transmission occurs in the country. Further evaluation of quantitative rapid diagnostic tests for G6PD is warranted, especially for females in whom the random X chromosome inactivation may affect the interpretation of qualitative tests.

## Abbreviations

ACT: artemisinin-based combination therapy
G6PD: glucose-6-phosphate dehydrogenase
CNTS: Centre National de Transfusion Sanguine (National Center for Blood Transfusion)
EDTA: ethylenediaminetetraacetic acid
G6PD: glucose-6-phosphate dehydrogenase
RFLP: restriction fragment length polymorphism
